# A frequency analysis of filterbank initialisation and noise augmentation for LEAF

**DOI:** 10.1038/s41598-026-49403-4

**Published:** 2026-04-25

**Authors:** Manuel Milling, Andreas Triantafyllopoulos, Simon D. N. Rampp, Björn W. Schuller

**Affiliations:** 1 CHI – Chair of Health Informatics, MRI, Technical University of Munich, Munich, 81675 Germany; 2 MCML – Munich Center for Machine Learning, Munich, Munich, 80333 Germany; 3 MDSI – Munich Data Science Institute, 85748 Garching, Germany; 4 GLAM – the Group on Language, Audio, & Music, Imperial College London, London, SW7 2AZ UK

**Keywords:** Speech signal analysis, Computer audition, Filterbanks, Learnable frontends, Deep learning training dynamics, Electrical and electronic engineering, Computational models, Machine learning, Computational science

## Abstract

Differentiable frontends, such as the LEArnable Frontend (LEAF), have drawn increasing interest from the computer audition (CA) community combining the rigour of traditional signal processing techniques with the flexibility and potential of end-to-end deep learning approaches. Concretely, they promise the ability to automatically learn task-specific features, resulting in both higher performance and better interpretability of CA applications. With the adaptability of LEAF’s parameters being questioned in recent literature, we further dig into the reasons why LEAF does not adjust its parameters. We thus perform a detailed analysis investigating the effects of filterbank initialisation for LEAF in a wide, previously unmatched range of computer audition tasks, namely speech recognition, speech emotion recognition, acoustic scene classification, and bird activity detection. In line with literature, we report that performance stays constantly high irrespective of filterbank initialisation, so long as it covers the entire frequency spectrum, in which case adaptation is minimal. Crucially, however, a filterbank initialised with all frequency bands equally does change its centre frequencies and bandwidths, yet remains with a lower performance. This effect is seemingly independent of how information is spread across frequencies, as we confirm in an additional set of experiments with controlled frequency distributions. This points towards the critical role of initialisation and the inductive bias of LEAF and manifests concerns about the adaptability and interpretability of LEAF across many settings. The code for our experiments is publicly available under https://github.com/millinma/LEAFFrequencyAnalysis.

## Introduction

Over the last two decades, *deep learning* (DL) has become the dominant driver in pushing the performance limits of virtually all computer audition tasks^1,2^. Despite their “black-box” character resulting from the large parameter space, model inputs are still mostly audio features based on traditional signal processing techniques^3–6^. Typical examples are Mel-spectrograms^7^ or *Mel frequency cepstral coefficient* (MFCCs)^8^.

With the advent of DL, many attempts have tried to replace traditional audio features with *end-to-end* learning, with architectures acting on the raw signal^9–15^. For instance, these can comprise a mix of 1-D convolutional layers and recurrent layers with *long short-term memory* (LSTM) cells. Such models do not carry the inductive biases of traditional features and can more freely explore an unconstrained parameter space to reach the best solution (though, in practice, they have been shown to converge to traditional feature sets^16^).

A ‘middle-ground’ solution is the use of audio-specific *frontends* – differentiable modules which draw inspiration from traditional *digital signal processing* (DSP) algorithms but make their parameters learnable^17^. Amongst a larger set of differentiable audio frontends^18–20^ the most popular versions are SincNet^21^ and *learnable frontend* (LEAF)^22^, both of which rely on parametrised band-pass filters. This idea embodies an exciting combination of end-to-end learning and prior expert knowledge, thus promising to enable on the one hand the full power of DL by making all relevant parameters learnable – including those used for feature extraction. On the other hand, it still leverages fundamental principles from traditional signal processing. The network is thus expected to adapt filter parameters to fit the information distribution across different tasks.

Our focus here is on LEAF, as it has been gaining increasing attention due to its effectiveness on a variety of computer audition tasks^22–25^. However, recent work by Meng et al.^26^ investigated which parameters are actually changed during training when using a LEAF frontend for different speech datasets. They concluded that, of the three learnable blocks of LEAF, only the *per-channel energy normalisation* (PCEN) (i.e., a frequency-specific normalisation factor) changed to a significant extent, but neither the Gaussian lowpass pooling (i.e., a learnable downsampling element) nor – most importantly – the learnable filterbank (i.e., its centre frequencies or bandwiths). Given that the malleability of these parameters is used as a key argument for the success of LEAF^22^, this finding brings up the question: *Why does LEAF not adjust these parameters?*

In this contribution, we dive deeper into this question. Crucially, the initialisation of the LEAF filterbank is purposefully chosen with an implementation of the standard Mel-scale^22^. One reasonable hypothesis is, therefore, that the Mel-filterbank initialisation of LEAF is already a nearly optimal filterbank for speech tasks and that this is why it did not show any changes during training. The Mel-filterbank is based on the Mel-scale, which was designed to mimic the frequency perception of the human ear and may thus be a well-designed general-purpose tool to process frequencies across the full frequency range of human hearing and, in particular, to process speech. However, when looking at more specific tasks individually, such as the analysis of bird-to-bird communication, there may be better-suited non-linear perceptual frequency scales to which a learnable filterbank may converge, e.g., one mimicking a bird’s auditory system. Yet, when we confront the model with human-annotated data, we can not avoid introducing a certain human bias into the learning process of LEAF, further impeding the estimation of an optimal filterbank setting.

A first study investigating how LEAF behaves with different filterbank initialisations was performed in^27^, once more confirming a lack of parameter adjustment in the filterbank independent of the initalisation. Beyond that, authors report the highest performance with a linearly initialised filterbank, suggesting that, in fact, a Mel-based initialisation may not be optimal. While this finding once more showcases the lack of promised adjustability in the learnable frontend, the study is still only of limited expressiveness regarding the learning capabilities of LEAF: Given that the chosen filterbank initialisations cover the whole frequency range, the backbone model still has access to all frequencies such that the frontend does not necessarily have to adapt to learn the task.

With that in mind, we investigate the training dynamics of LEAF in a rigorous study across a variety of different datasets and different learning settings with the following probes: Firstly, we explore whether the learning of centre frequencies and bandwidths differs between speech-based and other computer audition tasks. We thereby aim to validate the reported learning behaviour of LEAF’s filterbank in^26,27^ in a more extended setting of datasets, including both a paralinguistic (as in^26^) and a linguistic (as in^26,27^) speech task, a dataset containing bird vocalisations (as in^27^), as well as a more general and complex CA task in acoustic scene classification, containing a variety of different audio cues in corresponding frequency ranges. We do so both in terms of parameter changes and the performance of trained model states.

We follow this train of thought with a more controlled series of experiments, in which we systematically corrupt the speech recognition dataset by filtering the signal in certain frequencies and adding frequency-dependent noise to see whether LEAF adjusts its filterbank range to focus on information-containing frequencies. In general, our probing experiments provide a more detailed understanding of how LEAF behaves during training, and particularly elucidates the interplay between initialisation and informational content in the different frequency ranges.

## Methods

### Learnable frontend (LEAF)

LEAF^22^ is designed to replace explicit pre-processing steps for raw audio files (like the extraction of frequency-based features in the form of spectrograms) by performing similar processing steps through parametrised operations in the network. The output of this learnable frontend, having a similar form of representations as spectrograms, then serves as input to a backend neural network – often a *convolutional neural network* (CNN) – and the parameters of the frontend are learnt jointly with the parameters of the backend. For this purpose, LEAF takes in a one-dimensional waveform signal with *T* samples and transforms it into an output of size $M\times N$, with a number of *N* filters and *M* time windows, following three steps: The first step, which shall be the focus of this work, is the convolution of the input signal with a set of *N* 1-D Gabor filters $\phi _n$, followed by a squared modulus operator. The Gabor filters^28^ are characterised by the bandwidth $1/\sigma _n$ of their Gaussian kernel and the centre frequency $\eta _n$ of their sinusoidal signal, with both centre frequency and bandwidth being learnable parameters. The convolution of the input signal and the Gabor filters thus produce an output of $T\times N$, which can be seen as the signal passing through *N* bandpass filters. The second step in the LEAF pipeline is a Gaussian lowpass filter with a learnable bandwidth and a fixed window and hop size, which reduces the resolution of the signal from $T\times N$ to $M\times N$. Finally, additional learnable parameters are introduced through a PCEN, the behaviour of which during training has been analysed before^26^.

As a backend for classification, we employ EfficientNet-B0^29^, a lightweight CNN architecture with approximately 4 million parameters, which has often served as a backbone for LEAF in other studies^22,26,30^. The number of output neurons is adjusted to the number of classes in each *computer audition* (CA) task.

### Filterbank initialisation

Our main point of investigation in this contribution is on the interplay of the Gabor filter parameters and the training for various computer audition tasks. For that purpose, we explore different initialisations of the Gabor filterbank to investigate how LEAF adjusts to different starting points or prior knowledge. Amongst other things, we aim to better understand whether different filterbanks are preferable across tasks and to what extent LEAF is able to converge to them. In addition to the initialisations explored in^27^, we also include a particularly suboptimal constant initialisation, which gives all channels of LEAF access to the same frequency range.

**Mel-scale**: The standard initialisation of LEAF’s Gabor filters follows centre frequencies and bandwidths initialised according to the Mel-scale^7^. With a long history of research, the Mel-scale is an attempt to quantify the sensitivity of human hearing perception in different frequency ranges. It is characterised by a higher frequency resolution in the lower frequencies and lower density in the higher frequencies. The transformation for a given point *m* on the linear Mel-scale to a corresponding frequency $\eta$ (in Hz) follows an exponential distribution^7^. The initialisation covers 40 filter bands with equidistant centre frequencies across the Mel-scale, ranging from $65\,\textrm{Hz}$ to $7800\,\textrm{Hz}$. Bandwidths are correspondingly initialised around each centre frequency, such that they roughly cover a range from the previous to the following centre frequency.

**Bark-scale**: The Bark-scale is a psychoacoustic scale, sharing some similarities with the Mel-scale but with the main focus on perceived loudness across the frequency spectrum. We once again derive an initialisation for LEAF filters using equally spaced frequencies, in a similar frequency range as the Mel-scale, with bandwidths once more approximately spanning from one centre frequency to the next. Our conversion to Hertz (including error corrections) follows Traunmüller^31^.

**Linear**: In contrast to the previously mentioned psychoacoustics-informed scales, we additionally define an initialisation covering the same frequency range, but with equal distances of centre frequencies sampled on a linear scale, thus omitting any prior bias from human hearing. The bandwidths are defined with a constant value of 420 Hz, slightly below the median bandwidth of the Mel-initialisation, thus offering insights into whether smaller bandwidths for smaller frequencies are learnt automatically.

**Constant**: As the final type of initialisation, we choose a constant centre frequency of 684 Hz, right at the centre of the Mel-scale and the same constant bandwidth as in the linear case. This resembles effectively one identical bandpass filter across all channels. LEAF thus needs to adjust its parameters to get access to information in different frequency ranges.

### Datasets

Our selection of datasets features a high variety of auditory cues and target tasks, showing considerable differences in information distribution across frequencies, which in turn might favour different frequency distributions in the filterbanks. We combine tasks covering previous related research in the form of linguistic and paralinguistic speech tasks, as well as a bird recognition task. We further extend our experiments to a more diverse acoustic scene classification task containing a large variety of audio events. The datasets are publicly available for research. The experiments do not pose ethical concerns conflicting the conditions under which the data sets are released. All audio samples are resampled to $16\,\textrm{kHz}$, if necessary, to match the input requirements for a common LEAF model. All splits are reproducible as we used and stored fixed random seeds to create splits where necessary, which are available in our repository.

***Speech recognition*** **(SR):** The Speech Commands dataset^32^ is a common benchmark dataset for SR, with data splits provided by *torchaudio*. The task is to assign clean speech recordings of around $1\,\textrm{s}$ length to one out of 35 keywords.

***Speech emotion recognition*** **(SER):** The FAU-AIBO corpus^33,34^ was introduced as the first ever SER challenge and has a stronger focus on paralinguistic information in speech, giving insight about the subject’s emotions. We choose the 2-class version of the task, thus a classification of utterances to either of 2 emotion classes. The dataset is split according to the *Interspeech 2009 Emotion Challenge*^35^.

***Acoustic scene classification*** **(ASC):** To investigate the behaviour of LEAF beyond speech-based tasks, we opt for Task 1 of the DCASE2020 challenge. The $10\,$s long audio chunks have to be classified to one out of 10 acoustic scenes and contain environmental noises, such as “natural” animal sounds, but also machine sounds, often engineered towards a low auditory disturbance of the human hearing^36^; we use the official training/evaluation splits of the challenge data.

***Bird activity detection*** **(BAD):** Finally, the BAD task contains audio with the least adaption to human hearing, as bird vocalisations are believed to be evolutionary developed for bird-to-bird communication and are evidently in higher frequency ranges than the majority of human communication^37^, which was also the justification to include a bird-related task in^27^. Additionally, LEAF has explicitly shown to outperform non-learnable frontends for bird activity recognition^23^. We use the datasets from the DCASE 2018 Bird Audio Detection challenge^38^, comprising data from three distinct source. As test set, we use the *“warblrb10k”* part of the data, while we do a random 75%−25% split of the other two sources for train and validation.

### Noise and bandpass filter augmentations

In order to further assert control over the frequency profile of our data in our second line of experiments, we incorporate three types of augmentation techniques, one limiting the information content of the frequencies and two adding frequency-specific noise signatures to the original audio.

**Bandpass filtering**: We begin with bandpass filtering using 2^nd^ order Butteworth filters that aim to limit the frequency content of the signal beyond a certain range of frequencies. Specifically, we design 10 bandpass filters with their centres and bandwidths following the Mel-scale within the same frequency range as described for the Mel initialisation. We apply one bandpass filter at a time during the experiments, making sure that only frequency content within the chosen bandpass filter remains. This aggressive removal of frequency content serves to limit the exploration landscape for LEAF; our hypothesis is that the model will adapt to the limited bandwidth by focusing on the available frequencies.

**Low-passed noise**: We add low-passed noise to the data, where we start from uniform white noise and low-pass it with 2^nd^ order Butteworth filters with cutoff frequencies being the centres of the 10-part Mel-scale defined above (this is inspired by pink noise);

**High-passed noise**: Finally, we add high-passed noise to the signal, where we start from unfiltered broadband white noise and high-pass it in similar fashion (this is inspired by blue noise). Note that instead of pink/blue noise we opted for these alternative definitions of pink and blue noise because we wanted zero noise (rather than attenuated noise) in the higher/lower frequencies.

### Experiments

All experiments are performed with the same model architecture consisting of a LEAF frontend and EfficientNet-B0, only deviating in the number of neurons in the classification layer and their filterbank initialisation, on all four CA tasks. We opt for a unified training setting across datasets and initialisations. We train all models for 50 epochs with balanced cross-entropy loss, Adam optimiser with a learning rate of $3\cdot 10^{-4}$, and a batch size of 32. During training, the model is evaluated on the validation data after every epoch. The final evaluations on the test data are then performed for the model states with the best validation performance in each training run. The code is based on the autrainer package^39^ and is publicly available. We note that from the 48 experiments only five models showed improvement with respect to the development loss in the last three epochs of training, with the majority of experiments reaching their lowest development loss long before the final epoch. Overall, we thus assume that most models have reached a reasonable level of convergence, allowing us to further analyse their trained model states. Training was performed on a NVIDIA GeForce RTX 3090 with per-epoch training times of approximately 6:37 min (SR), 1:22 min (SER), 8:29 min (ASC), and 7:33 min (BAD).

## Results

### Effects of filterbank initialisation

**Table 1:** Unweighted average recall (UAR)/unweighted average F1 Score (UAF1) of the LEAF model on the datasets with different initialisations. Results are reported with mean ± standard deviation across three random seeds. The best results per initialisation are highlighted in bold.

**Initialisation**	**Speech recognition**	**Speech emotion recognition**	**Acoustic scene classification**	**Bird activity recognition**
Constant	.877 $\pm .003$/.874 $\pm .003$	.610 $\pm .010$/.605 $\pm .012$	.407 $\pm .006$/.397 $\pm .007$	.576 $\pm .025$/.417 $\pm .075$
Linear	.926 $\pm .002$/.925 $\pm .003$	$\mathbf {.660}$ $\pm .009$/$\mathbf {.639}$ $\pm .015$	.468 $\pm .008$/.461 $\pm .017$	.809 $\pm .014$/.751 $\pm .021$
Bark-scale	.934 $\pm .002$/.934 $\pm .003$	.654 $\pm .010$/.636 $\pm .007$	.473 $\pm .007$/.468 $\pm .006$	$\mathbf {.813}$ $\pm .008$/$\mathbf {.765}$ $\pm .005$
Mel-scale	$\mathbf {.935}$ $\pm .002$/$\mathbf {.937}$ $\pm .001$	.652 $\pm .004$/.633 $\pm .004$	$\mathbf {.485}$ $\pm .007$/$\mathbf {.481}$ $\pm .007$	.806 $\pm .018$/.753 $\pm .009$

Table 1 summarises the results of our first line of experiments, which does not include any previously introduced noise and filter augmentations. We report unweighted average recall (UAR) and unweighted average F1 score (UAF1), both of which are calculated on a class-level – F1 score being the harmonic mean of precision and recall – and are then averaged across all classes with equal contribution of all classes independent of potential class-imbalance. Table 1shows that LEAF is able to learn all of the CA tasks with virtually any filterbank initialisation, even though the performance is clearly impacted if a constant initialisation is chosen. Despite differences in the absolute values of the two performance measures, the overall tendencies when comparing across different intialisations are identical. While the Mel-scale seems to show a slightly favourable performance overall, both the Bark-scale and the linear initialisation show no major deviations from it. A constant initialisation, on the other hand, shows by far the worst performance. Interestingly enough^27^, report a favourable performance of a linear over Mel-scale and bark-scale initialisation, which is a trend we cannot observe in our experiments.

**Fig. 1:**
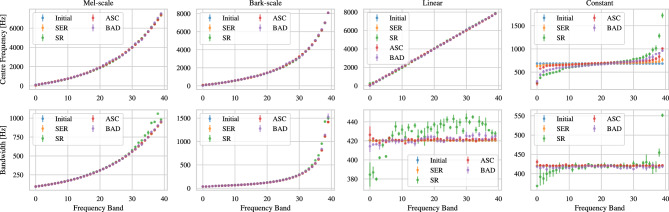
Overview of the centre frequencies (top) and bandwidths (bottom) after training. Each column shows different parameter initialisations in blue and the parameter values after training on the respective datasets.

Some insight into the inner workings of the filterbanks can be drawn from Figure 1. It is noteworthy that neither of the three well-performing initialisations, namely Mel-scale, Bark-scale, and linear, show a large change in centre frequencies. This is in-line with the results found in^26^ that the Mel-initialised LEAF does not change filterbanks by a large amount. We note that LEAF’s Gabor filters essentially operate as bandpass filters. It seems, therefore, that some access to the information present in the entire frequency range is enough to learn a good classifier for all CA tasks, but that the network does not need to learn any features more advanced than that. A possible explanation would be that any unfavourable information present in any of the learnt frequency ranges can be mitigated through the PCEN, which has been shown to change during training^26^.

The clear exception to both the good performance and the lack of adaptation is seen with the constant initialisation. As per its motivation, LEAF has only access to a small part of the frequency range at initialisation, namely $684 \pm 420$ Hz. LEAF seems to partially circumvent this problem by changing its centre frequencies and bandwidths. Interestingly, the parameters at the end of training follow the same general pattern of a slight S-curve, contrasting the other scales, however to varying extent for the different tasks.

Similar to centre frequencies, most bandwidths are almost unaffected, with the exception of SR, where a slight tendency to lower bandwidths for lower frequencies and higher bandwidths for higher frequencies can be observed. This is particularly the case for the constant and linear initialisation, both of which act from a constantly initialised bandwidth, but can also be observed to some extent in the Mel- and Bark-scale initialisation. One possible reason for the adaptability of the bandwidth for the SR dataset may lie in the generally high performance on this 35-class classification problem across initialisations (c.f. Table 1); a particularly easy dataset may reduce gradient noise and therefore ease parameter changes.

Overall, it seems thus possible that the model is converging to an approximation of the Mel-scale, yet with a high “inertia” or fuzzy gradients keeping the model from striving too far away from its initialisation. For instance, filters trained on the SR task show the widest coverage in the frequency spectrum, reaching over 1500 Hz, considering the range of centre frequencies and corresponding bandwidths, whereas other tasks remain below 1000 Hz, even though the information relevant for spoken word recognition is expected at lower ranges than for SER or – especially – BAD. This counterintuitive finding illustrates how LEAF might not be converging to a state that exploits the relevant frequency information in the same way humans do, at least if this bias is not baked into it through initialisation (e.g., using the Mel-scale).

In this context, it is also worth noting that we observed a slower convergence of the training loss for the constant initialisation compared to other initialisations with overall three of the five model states showing development improvements in the last three epochs being attributed to this initialisation. It seems therefore possible that, with longer training, this gap can be further closed, yet it seems unlikely that under the current training setting the constantly initialised model would catch up with the other types of initialisation (in terms of parameter convergence or performance) simply through continued training. On the other hand, in the case of the SER task, the smallest of all investigated datasets, all models tend to overfit quickly and the best validation performance is already achieved after few epochs. This might have affected our results with respect to the amount of change seen for the different tasks.

### Impacts of noise and bandpass filter augmentations

Given our observation that the centre frequencies of LEAF seem biased towards converging to an ascending curve irrespective of their initialisation, we investigate applying the previously introduced filter and noise augmentations to our data that intentionally limit/corrupt the frequency content. We focus for these experiments on the SR dataset for which we observed the largest overall changes in the original training.

**Fig. 2:**
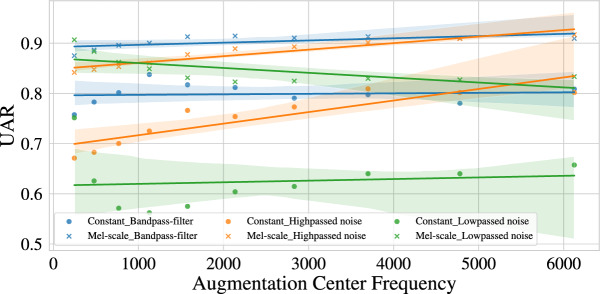
Performance of LEAF with constant and Mel-scale initialisation on the SR dataset. The unweighted average recall (UAR) is displayed in relation to the corresponding frequency range for different augmentation types.

We end up with a total of 30 versions of the SR dataset (10 per type of augmentation). For all three cases, we train a LEAF model starting from the constant initialisation and Mel-initialisation. The training is analogous to the previous experiments but with an adjusted learning rate of $10^{-4}$, which turned out favourable for the fast-converging SR task and, due to limitations in computational resources, a reduced amount of 20 epochs.

Figure 2 shows the performance of LEAF with Mel-scale and constant initialisation for the three types of data augmentations with respect to the centre/cutoff frequency of the augmentation. We note that the overall performance is lower than using the original, unaugmented datawith 0.934 and 0.877 for Mel-scale and constant initialisation, respectively. This is expected given that prior work on corrupting speech with background noise^40^ or coding^41^ has shown a deterioration in performance.

Surprisingly, the performance of the bandpass augmentation stays very stable for both initialisations, independent of the frequency range to which the data is restricted. On the other hand, the low-passed noise seems to be always detrimental to performance, with greater effects in the higher frequency ranges, at least in the case of the Mel-scale. The high-pass noise shows a clear trend in both initialisation cases achieving better performance for higher centre frequencies.

Similar to Table 1, the Mel-initialisation shows a higher performance than the constant initialisation throughout. This is unexpected, as the bandpass filtering removes any additional information that the Mel-initialisation could utilise. Especially for the case where the filter range covers the constant initialisation (684 Hz), the available bandwidth should suffice for the constant initialisation.

**Fig. 3:**
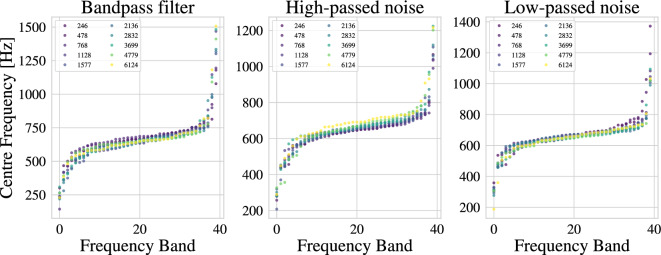
Centre frequencies learnt by LEAF on augmented data. Depicted are the centre frequencies after training LEAF with constant initialisation on the speech recognition dataset with different augmentation parameters. Each plot represents a different augmentation type.

Figure 3 further shows the impacts of augmentation on the different LEAF filters in the case of a constant initialisation (only centre frequencies are shown due to space limitations). Remarkably, the filters follow the same general trend of converging to an S-shaped curve that covers a broad range of frequencies – despite the fact that these frequencies have been filtered out (bandpass) or masked by noise. For example, in the case of a bandpass filter centred on the lowest frequency (246 Hz), we see the upper bands converging to frequencies over 1000 Hz, where no information is expected to be found. Similarly, for noise augmentations, this shows a strong bias of LEAF filters towards that shape even if no information is present in the frequencies that it converges to.

## Discussion & conclusion

**Summary:** Collectively, our results show that the learnable LEAF filterbank has a high resistance to changing its parameters with the centre frequency having the tendency to converge to an S-shaped curve, irrespective of whether information is present in the range of frequencies it covers, as shown by our augmentation experiments. This may explain why previous works did not see much change in the filterbank values with the Mel-scale having access to the whole frequency range and LEAF thus having little incentive to move away from it^26,27^. Our initialisation experiments show that the choice of the Mel-scale is more-or-less arbitrary, with a linear initialisation performing similarly well, which in fact showed even higher performance in^27^. The interesting corollary is that the use of LEAF as a novel inductive bias for audio architectures might not be leading to wildly more exotic features than the traditional Mel-filterbanks typically employed by most works.

**Limitations:** An open question remains why the incentive of LEAF to learn its filterbank parameters is limited, despite the apparent benefit of well-adapted parameters. Reasons for this could either be generally small gradients for centre frequencies or bandwiths resulting from the network design itself or a more complex interplay between the LEAF frontend and the backend classifier, as well as their combined loss landscape. The latter might be explained by frontend parameters being stuck in a local minimum and the learning trajectory in turn only being driven by parameter changes in the backend model. The understanding of training dynamics in deep neural networks is still a very active field of research, tackling questions, like how initialisation impacts the solution spaces, what the role of different layers are for the learning of neural networks^42^, or what makes models generalise, with only few works studying the specifics of computer audition^43,44^.

**Conclusion:** We investigated the learning behaviour of the LEAF frontend with respect to centre frequencies and bandwidths in various contexts for CA tasks. We discovered that the performance of LEAF is stable as long as the filterbank initialisation covers all relevant frequency ranges and it does not majorly adjust its parameters in this case. If the initialised model however has only access to a single initialisation across filters, centre frequencies and bandwidths can be learned in a similar manner, but not to the same extent for different ASC datasets and the model performance does not catch up with full-range initialisation. The learning process of filterbank parameters is –interestingly enough– not majorly impacted by the corruption or removal of certain frequencies.

**Future Avenues:** Under standard training settings, it seems evident at this point that LEAF fails to offer promised benefits regarding interpretability based on a desired adaptation of (filterbank) parameters to task-specific frequency ranges. A critical role of early layers in the overall model performance despite small changes during training has been reported before in DL research^42^. To overcome this, future steps should be investigated to manipulate the training procedure of LEAF to encourage stronger changes in the filterbanks. One likely direction through which this could be realised are layer-adjusted learning rates or sharpness-aware optimisation^45^. These methods might help overcome small gradients in the deep combination of LEAF and backbone model and lead to more robust solutions. Similarly, parameter changes might be explicitly rewarded through regularisation terms in the loss function, potentially in combination with iterative training designs^46^.

## Data Availability

All datasets on which this study is based are publicly available. Three datasets can be downloaded automatically via the provided code. One dataset can be accessed upon request; please contact the corresponding author to do so. The code for precise reproduction of the results including a detailed description is available under https://github.com/millinma/LEAFFrequencyAnalysis.
